# Structure of the African swine fever virus major capsid protein p72

**DOI:** 10.1038/s41422-019-0232-x

**Published:** 2019-09-17

**Authors:** Qi Liu, Bingting Ma, Nianchao Qian, Fan Zhang, Xu Tan, Jianlin Lei, Ye Xiang

**Affiliations:** 1 Beijing Advanced Innovation Center for Structural Biology, Beijing Frontier Research Center for Biological Structure, Center for Infectious Disease Research, Department of Basic Medical Sciences, School of Medicine, Tsinghua University, Beijing, 100084 China; 2 Tsinghua University-Peking University Joint Center for Life Sciences, Beijing, China; 3 Beijing Advanced Innovation Center for Structural Biology, Beijing Frontier Research Center for Biological Structure, School of Pharmaceutical Science, Tsinghua University, Beijing, 100084 China; 4 Beijing Advanced Innovation Center for Structural Biology, Beijing Frontier Research Center for Biological Structure, School of Life Science, Tsinghua University, Beijing, 100084 China

**Keywords:** Electron microscopy, Cryoelectron microscopy

Dear Editor,

African swine fever (ASF) caused by the African swine fever virus (ASFV) infection is a highly contagious and lethal disease of domestic pigs. Recent spread of the virus to China,^1^ the biggest pork-consuming country, has caused huge economic loss. So far there is no effective way to prevent the spread of the virus. Vaccine for protection from ASFV infection or effective treatments to cure ASF is urgently needed.^2^ ASFV is the only member of the family Asfarviridae that belongs to the group of nucleocytoplasmic large DNA viruses (NCLDVs). Early studies showed that the virion has a complex structure with multiple membrane and protein layers.^2^ The outmost protein shell of the virion is an icosahedral capsid mainly assembled by the viral gene *B646L* encoded protein p72. The major capsid protein (MCP) p72 is the most dominant structural component of the virion and constitutes about ~31%–33% of the total mass of the virion,^2^ making it one of the major antigens detected in infected pigs.^3^ Monoclonal antibodies recognizing p72 were shown to neutralize virulent ASFV isolates.^4^ Here we report the chaperon-aided folding of p72 and the cryo-electron microscopy (cryo-EM) structure of p72 at a resolution of 2.67 Å.

Early studies showed that B602L of ASFV is required for the formation of the icosahedral capsid^5^ and functions to aid the production of trypsin-resistant p72.^6^ Our results showed that the expression of p72 alone in HEK293F cells resulted in formation of soluble aggregates of p72 (Supplementary information, Fig. S1a). Correctly folded and assembled p72 could be obtained only when p72 and B602L were co-expressed in HEK293F cells (Supplementary information, Fig. S1b–e). We then determined the structure of p72 at a resolution of 2.67 Å using cryo-EM single-particle 3D reconstruction (Supplementary information, Figs. S2, 3 and Table S1). The structure of p72 shows that three p72 molecules form a stable trimer spike of ~85 Å in height and ~85 Å in diameter (Fig. 1a). The p72 monomer adopts a double jelly-roll structure, which is common for the capsid proteins of many other icosahedral viruses.^7^ The two (N- and C-terminal) jelly-roll β-barrels constitute the pseudo hexagonal base of the trimer, and the insertions in between β-strands of the jelly-roll barrels constitute the protruding top of the trimer (Fig. 1a). Every single jelly-roll β-barrel is composed of two opposite β-sheets, each consisting of four antiparallel β-strands. The eight sequential β-strands along the polypeptide chain are named as B, C, D, E, F, G, H and I, respectively, with BIDG forming one β-sheet and CHEF forming the other β-sheet (Fig. 1b, c). The ASFV p72 has large insertions between strands D and E (DE_N_ loop), strands F and G (FG_N_ loop), strands H and I (HI_N_ loop) of the N-terminal jelly-roll barrel, and between strands D and E (DE_C_ loop) of the C-terminal jelly-roll barrel (Fig. 1b, c). The DE_N_ loop has 79 residues folding into a ~53 Å long protruding hairpin with five β-strands (DE_N_-β1-5) and one α-helix (DE_N_-α) (Fig. 1b, c). The FG_N_ loop has 122 residues folding into two α-helices (FG_N_-α1-2) and two β-strands (FG_N_-β1-2). The HI_N_ insertion consists of 49 residues folding into three β-strands (HI_N_-β1-3) and one α-helix (HI_N_-α). The DE_C_ loop consists of 64 residues folding into four β-strands (DE_C_-β1-4) and one α-helix (DE_C_-α) (Fig. 1b, c). The strands DE_N_-β4, HI_N_-β2-3 and DE_C_-β2-3 form a five-stranded antiparallel β-sheet. Three β-sheets assemble into blades to form a screw propeller-like cap on the top of the trimer spike (Fig. 1a, c). The blades are connected to the jelly-roll base through strands DE_N_-β1-3, 5, HI_N_-β1 and DE_C_-β1, 4. Below the screw propeller-like cap is another layer of three twisted β-sheets constituted by twelve β-strands (central β-layer). Each β-sheet is assembled by the intertwined strands FG_N_-β1 and FG_N_-β2 of one monomer and strands DE_C_-β1 and DE_C_-β4 of a neighboring monomer (Fig. 1a, c). The two β-strands of the FG_N_ loop form a β-hairpin structure going across the gap between two neighboring monomers. The distal tip of the β-hairpin is located in between the two neighboring β-sheet blades of the screw propeller and is highly disordered (Supplementary information, Fig. S4).

**Fig. 1:**
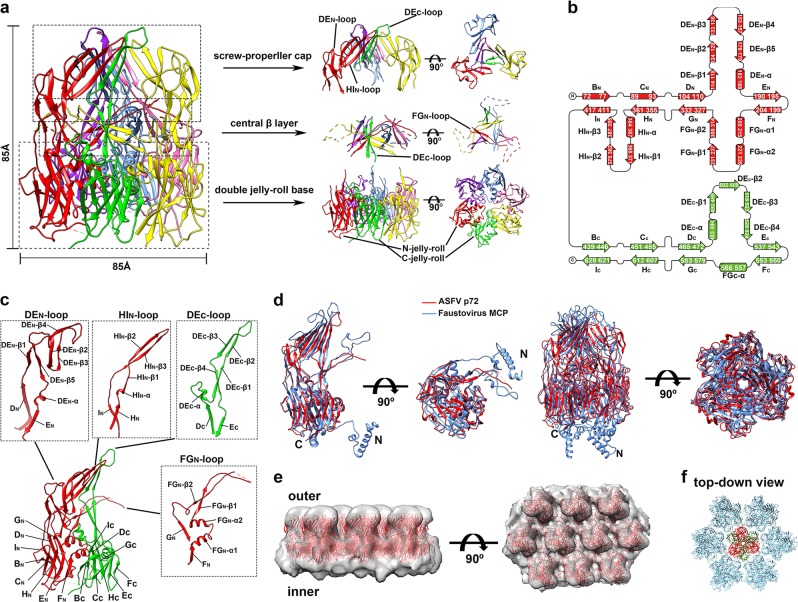
The p72 structure. **a** Ribbon diagrams showing the structure of the p72 trimer spike. The six jelly-roll barrels and the corresponding insertion domains are colored red, green, yellow, hot pink, cornflower blue and purple, respectively. The side (left) and top (right) views showing the details of the screw propeller-like top, the central β-layer, and the pseudo hexagonal base. **b** Diagrams showing the topology of p72. The N- and C-terminal jelly-roll barrels and the corresponding insertions are colored red and green, respectively. **c** Ribbon diagrams showing the structure details of the insertions. **d** Structure superposition of the ASFV p72 (red) and the faustovirus MCP (PDB: 5J7O)^8^ (cornflower blue). The N-terminal residues 1–70 of the ASFV p72 are disordered in the structure. **e** The fitted p72 trimers (red ribbons) in the cryo-EM map (EMD-8144)^8^ of the faustovirus capsid. The map is contoured at 0.7 σ and is shown as semi-transparent solid surface. **f** The fitted p72 trimer spikes show a zigzag arrangement of their pseudo hexagonal bases. The two jelly-roll domains of the p72 in the center is colored red and green, respectively. The p72 molecules around are all colored light blue

The interactions between the jelly-roll barrels are mainly hydrophobic and mediated by the helices FG_N_-α2 and FG_C_-α (Supplementary information, Fig. S5a). Inner cavities are observed between the top screw propeller-like assembly and the central β-layer, indicating no close interactions between the top structure layers (Supplementary information, Fig. S5b). The outer surface of the trimer spike is rich in charged residues (Supplementary information, Fig. S5c). No obvious glycosylation site was observed on the trimer spike (Supplementary information, Fig. S1f).

The structure of the ASFV p72 is highly similar to that of the faustovirus MCP (PDB: 5J7O)^8^ with a R.M.S.D. of 1.6 Å between 446 align C_α_ atom pairs (Fig. 1d). The sequence identity between the ASFV p72 and the faustovirus MCP, however, is 41% (Supplementary information, Fig. S6). Given the similarity between the major capsid proteins, we hypothesized that the capsid assemblies of the two large viruses could also be similar. Thus, we fitted the ASFV p72 structure into the EM density map (EMD-8144)^8^ of the faustovirus capsid (Fig. 1e). The results showed that the ASFV p72 structure could be well fitted into the faustovirus capsid EM density map with its screw propeller-like top protruding outwards and its pseudo hexagonal base anchoring on the inner virus surface. The fitting results indicated that the tops of the p72 trimers are well separated on the virus surface. The jelly-roll barrels in the pseudo hexagonal bases have close contacts in a zigzag arrangement (Fig. 1f). Anchoring of the p72 trimers is through the bottom of the trimer, which contains the N-terminal residues 1–70 of p72 and is highly disordered in our structure. In contrast, the N-terminal portion of the faustovirus MCP purified from virus particles is ordered and folds into two α-helices (Supplementary information, Fig. S7). Furthermore, helix fragments that do not belong to the capsid protein were observed in the structure of the faustovirus MCP and form helix bundles with the N-terminal helices.^8^ The helix fragments were hypothesized to belong to a yet to be identified viral surface protein that helps to anchor the faustovirus MCP. The corresponding N-terminal part of ASFV is highly similar to that of the faustovirus MCP (Supplementary information, Fig. S7) with 53.8% identical residues. Thus, p72 might use a similar mechanism to attach the inner virus surface and residues 1–70 of p72 could become ordered upon attachment. The outmost surfaces of the ASFV p72 and the faustovirus MCP, which might be related to specific binding of the receptors, show completely different features and greater variations compared to the hexagonal base (Supplementary information, Fig. S8).

Previous studies showed that p72 is a major antigen in infected pigs^3^ and is widely used as a marker for diagnosis of infection. However, none of the identified dominant linear or conformational epitopes^9–11^ are located on the most exposed top of the screw propeller-like cap (Supplementary information, Fig. S9a), which is constituted by the tips of the DE_N_, HI_N_ and DE_C_ loops (Fig. 1a–c). These tips have extensive interactions with each other through hydrophobic packing and hydrogen bonds, suggesting that conformational epitopes should be dominant for the most exposed top screw propeller-like structure (Supplementary information, Fig. S9b). We treated the purified p72 at 96 °C for 10 min. Native PAGE gel analysis of the treated protein showed that a large portion of the trimer spikes are still intact and stable (Supplementary information, Fig. S10), indicating the high thermal stability of p72 and suggesting the potential usage of p72 as a subunit vaccine.

Homolog search by using the jelly-roll barrels showed that the jelly-roll base of p72 is highly similar to those of other viral capsid proteins or scaffold protein (Supplementary information, Table S2). However, the top structures formed by the insertions are quite different among these viral proteins (Supplementary information, Fig. S11a). Of note, the three insertion domains of the ASFV p72 or the faustovirus MCP are gathered together in a closed state, whereas the top insertions observed in other double jelly-roll viral proteins are open and well separated (Supplementary information, Fig. S11). The Paramecium bursaria Chlorella virus (PBCV-1) is currently the only NCLDV with high-resolution structure available.^12^ The major capsid protein Vp54 of PBCV-1 has short insertions without forming a top domain at all. However, heavy glycosylations have been observed on the top of the Vp54 trimer.^13^ The p72 capsid assembly of ASFV was also generated by using the PBCV-1 capsid as a template. The results showed a similar zigzag arrangement of the jelly-roll base as has been generated with the faustovirus capsid EM map (Fig. 1e, f; Supplementary information, Fig. S12).

In summary, here we report the high-resolution cryo-EM structure of the ASFV major capsid protein p72 and showed that with the aid of B602L, three p72 molecules assemble to form a thermostable trimer that has a pseudo hexagonal base with six jelly-roll β-barrels and a screw propeller-like cap on the top of the pseudo hexagonal base. Our research could shed a light on development of vaccine and anti-viral drugs against ASFV.

## Supplementary information

Supplementary information

## Data Availability

The atomic coordinates and EM maps have been deposited into the Protein Data Bank (http://www.pdb.org) and the EM Data Bank with the accession numbers 6KU9 and EMD-0776, respectively.
