# Supplementary material for: Structure of the African swine fever virus major capsid protein p72
Source: Cell Res. 2019 Sep 17;29(11):953–5. doi: 10.1038/s41422-019-0232-x (PMC6889146; doi:10.1038/s41422-019-0232-x)
Supplement: Supplementary file 1 — Supplementary information [file 41422_2019_232_MOESM1_ESM.pdf]

Supplementary Information for

Structure of the African swine fever virus major capsid protein p72

Qi Liu[1], Bingting Ma[1], Nianchao Qian[3], Fan Zhang[3], Xu Tan[3], Jianlin Lei[2], Ye Xiang[1*]

[1]Beijing Advanced Innovation Center for Structural Biology, Beijing Frontier Research Center for Biological Structure, Center for Infectious Disease Research, Department of Basic Medical Sciences, School of Medicine, Tsinghua University, Beijing 100084

[2] Beijing Advanced Innovation Center for Structural Biology, Beijing Frontier Research Center for Biological Structure, School of Life Science, Tsinghua University, Beijing 100084

[3] Beijing Advanced Innovation Center for Structural Biology, Beijing Frontier Research Center for Biological Structure, School of Pharmaceutical Science, Tsinghua University, Beijing 100084

*To whom correspondence should be addressed: Y.X.: Tel.:+86-10-62772587; Email: yxiang@mail.tsinghua.edu.cn.

Materials and Methods

Recombinant protein production and sample preparation

The ASFV genes *B646L* and *B602L* (strain China Pig/HLJ/2018, GenBank: MK333180.1) were synthesized from the Qingnan company (Wuxi, China). The 3' region of *B646L* is used for the classification of different ASFV genotypes[1]. The strain Pig/HLJ/2018 was isolated from the primary porcine alveolar macrophages (PAMs) of an infected pig in the Heilongjiang province of China[2]. The genome sequence of China Pig/HLJ/2018 is highly similar to that of an ASFV isolate from Poland (Poland Pig/2017, GenBank: MG939588.1). Sequences of the *B646L* encoded p72 proteins are identical among the China Pig/HLJ/2018, Georgia/2007/1 (GenBank: FR682468.1) and other isolates from China (China Pig/SY/2018, GenBank: MH766894.1 and China Pig/Anhui/2018, GenBank: MK128995.1) (Figure S13). The *B646L* gene that encodes protein p72 is organized as a single open reading frame in the ASFV genome. However, the genomic organization of the homologous faustovirus MCP gene is quite complicate and distributed in a region of ~17000 bp with 11 exons and 10 introns[3].

The synthesized genes were cloned into the pCMV vector separately. An N-terminal 10×His-3×Flag tag was added to the recombinant p72 (gene product of *B646L*). An N-terminal 1×Flag and 1×Strep was added to the recombinant B602L. HEK293F cells were cultured in suspension at 37 °C with the 293-SIM medium (Product M293II, Sino Biological company) and 5% CO_2. For one-liter cell culture, the cells were transfected with a plasmid mixture containing 2 mg p72 plasmid, 2 mg B602L plasmid and 12 mg PEI at a cell density of 2×10^6 cells/ml. The transfected cells were harvested

48 hours post-transfection by centrifugation at 1000×g for 20 minutes. The cell pellet was resuspended by a buffer containing 20 mM HEPES at pH 7.4, 300 mM NaCl and cocktail protease inhibitors (Thermo scientific, Product A32965). The suspended cells were sonicated for 3 mins and the cell lysate was centrifuged for 20 mins at 160,000 rpm (JA 25.50 rotor, Beckman). The recombinant p72 protein in the supernatant were collected and applied to the anti-flag affinity beads (GeneScript Biological company, Product L00432). The bound protein was washed with the resuspended buffer twice and then was eluted from the beads with a buffer containing 0.1 mg/ml 3×flag peptide, 20 mM HEPES at pH 7.4 and 300 mM NaCl. The eluted sample was concentrated and further purified by a Superdex 200 increase 10/300 GL size exclusion column (GE healthcare, Product 28-9909-44) running in a buffer containing 20 mM HEPES (Amresco, Product 0511) at pH 7.4 and 300 mM NaCl (Amresco, Product 0241). The p72 protein from size exclusion column peak was concentrated and applied to a 10% - 30% w/v linear glycerol (sigma, Product G5516) gradient supplemented with 0.15% glutaraldehyde (sigma, Product G5882) for gradient fixation (GraFix). The gradient centrifugation was run for 13 hours at 240,000×g. Fractions containing p72 were collected and the glycerol was removed by buffer exchange through centrifugation with 20 mM HEPES at pH 7.4 and 150 mM NaCl. Overexpression of B602L was performed by using a similar procedure as having been described above for the coexpression of p72 and B602L. One liter of the HEK293F cells were transfected with 2 mg B602L plasmid and 6 mg PEI at a cell density of 2×10^6 cells/ml. The recombinant B602L was purified by using beads coated with anti-flag antibodies (GeneScript Biological

company, Product L00432). The protein was eluted with a buffer containing 0.1 mg/ml 3×flag peptide, 20 mM HEPES at pH7.4 and 300 mM NaCl. The eluted B602L was directly used for SDS-PAGE gel analysis (Figure S1e).

Purification of the recombinant p72 with size exclusion chromatography showed that p72 is an oligomer in solution, most likely a trimer based on its elution position (Figure S1c-d). SDS page gel analysis showed that the size exclusion chromatography peak contains only the p72, suggesting a chaperon role of B602L (Figure S1c-e). Similar chaperon-aided folding of viral capsid proteins has also been observed for other large dsDNA viruses[4].

Aliquots of 3.5 μl cross-linked p72 protein at a concentration of 0.4 mg/ml were applied to glow discharged holey carbon grids (Quantifoil, Cu 400 mesh, R1.2/1.3). The grids were blotted for 5.5 s in 100% humidity at 8 °C and were then immediately plunged into liquid ethane by using a Vitrobot Mark VI (Thermo Fisher).

Data Acquisition, image processing, model building and structure refinement

CryoEM images of p72 were collected at a nominal magnifications of 105,000 (which yields a calibrated pixel size of 0.5455 Å) on a 300 kV Titan Krios equipped with a GIF Quantum energy filter (slit width 20 eV) and a Gatan K2 Summit camera. Images were recorded as movie stacks under the counting mode in a defocus range of -1 μm to -3.5 μm. A total dose of ~ 50 electrons per $Å^2$ was used. AutoEMation2 was used for the data collection[5]. A total of 5317 movie stacks were collected. The frames in each movie stack were aligned, summed and 2× binned by using the program Motion

Cor2[6]. The CTF parameters of the micrographs were determined by using the program Gctf, which take local defocus variations into consideration[7].

A total of 4928380 particles were boxed by using Gautomatch. All the extracted particles were binned by a factor of 4 pixel × 4 pixel and subjected for reference-free 2D classifications by using RELION3.0[8]. The particles from classes with clear features were selected for further 3D classifications with C3 symmetry imposed (Figure S2). All the four classes from the classifications were processed for further 3D auto-refinements and the resultant maps from two classes show clear structural details and better resolutions than these from other two classes. These two classes were selected and particles from the selected class were used for further 3D classifications, respectively. Particles from the two of these further classified classes with similar structural details were combined and used for the final 3D auto-refinements that resulted in a 2.67 Å density map. The density map was applied with a negative B-factor of 104 $Å^2$ and corrected for the modulation transfer function (MTF) of the detector by using RELION. Particles from another class which showed different features at one distal end of the spike when comparing to the 2.67 Å structure were also selected and subject for 3D refinements, which result in a density map of 2.94 Å resolution. Comparisons between these two maps showed densities for three symmetry related additional helices at one distal end of the spike in the 2.94 Å map. However, the 2.67 Å map has better quality especially for the densities of residue side chains and was chosen for model building and structure refinements. The resolution was reported by the value at 0.143 of a Fourier shell correlation (FSC) curve calculated between two split datasets[9, 10] (Figure S3). The local resolution of the cryoEM density map was calculated by using ResMap[11]. The directional FSC curves were calculated by using the Remote 3DFSC Processing Server (https://3dfsc.salk.edu)[12] (Figure S3). The atomic model was built and adjusted by using COOT[13]. The model was refined by using PHENIX[14] cryoEM Real-space Refinement tool. Residues 1-70 at the N terminus, residues 249-303, 420-434 and 599-605 in the loop regions and residues 635-646 at the C terminus of the structure are highly disordered and not modeled in the final structure.

Search for homologous structures was performed by using the Dali server (http://ekhidna2.biocenter.helsinki.fi/dali/) [15].

Supplementary Figures

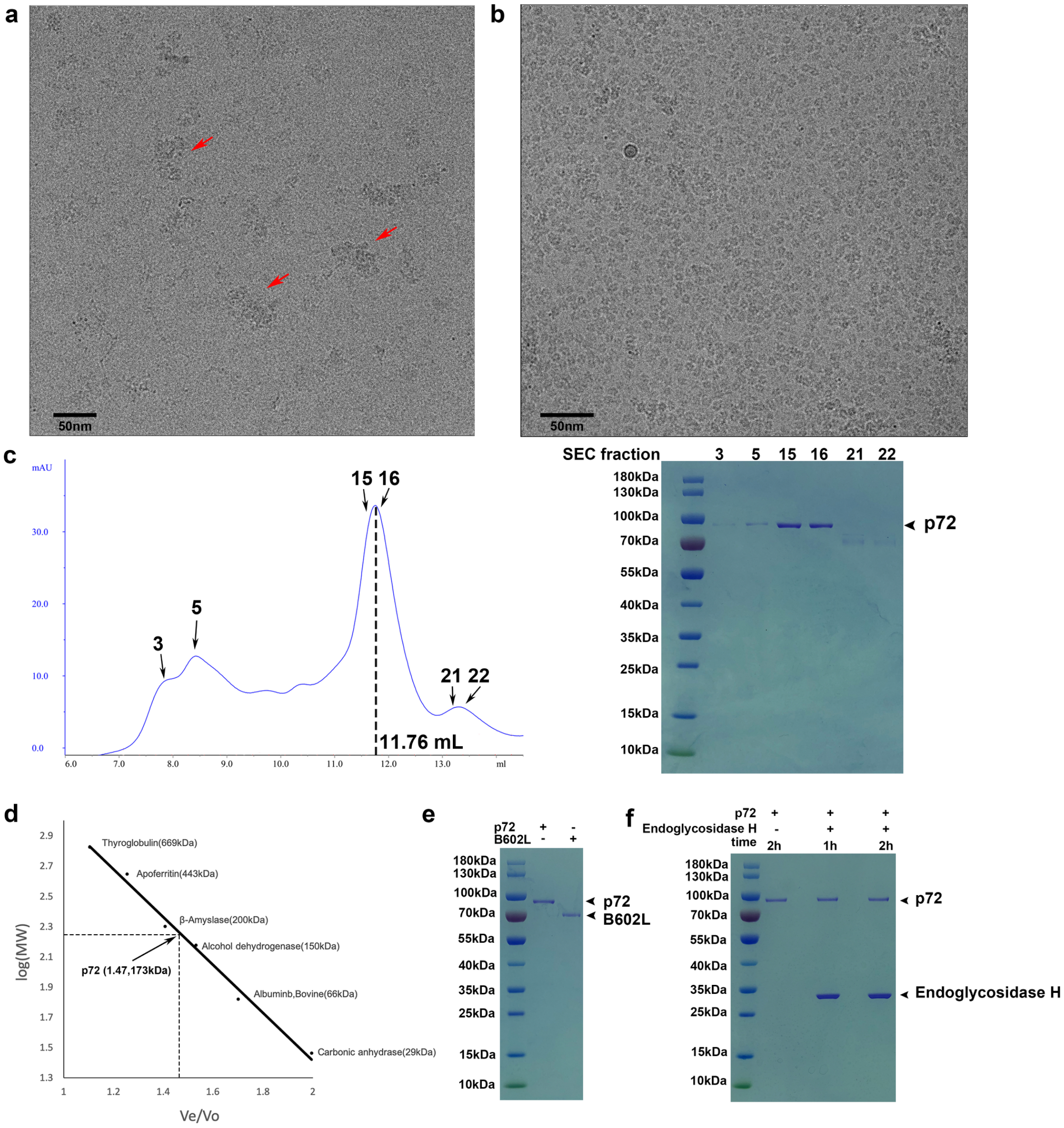

Supplementary information, Figure S1. Purification and characterization of the recombinant p72. (a) A representative cryoEM micrograph of the recombinant p72 produced without B602L. Red arrows indicated the soluble aggregations of p72. (b) A representative cryoEM micrograph of the recombinant p72 produced with B602L. Well folded particles are evenly distributed on the micrograph. (c) Size exclusion chromatography elution profile of the recombinant p72. The p72 elution volume is indicated by the dash line. SDS-PAGE gel analysis of the p72 elution profile is shown in the right panel. Position of the p72 band on the gel is indicated by the black arrow. (d) The calibration curve of the Superdex 200 column used for p72 purification. The estimated molecular weight (173 kDa) of p72 in solution is calculated based on the V_e/V_o value (1.47) of p72. (e) SDS-PAGE gel analysis of the purified p72 and B602L showing different migration of the two proteins on the gel, although with similar calculated molecular weights. Positions of p72 and B602L are indicated by black arrows. (f). SDS-PAGE gel analysis of the endoglycosidase H treated p72. The p72 protein was boiled at 100 °C for 10 minutes with 0.5% SDS. The heat-treated p72 protein was incubated with 500 units of endoglycosidase H (Promega, V4871) at 37 °C for one hour or two hours and was then used directly for the SDS-PAGE gel analysis. Positions of p72 and endoglycosidase H on the gel are indicated by black arrows.

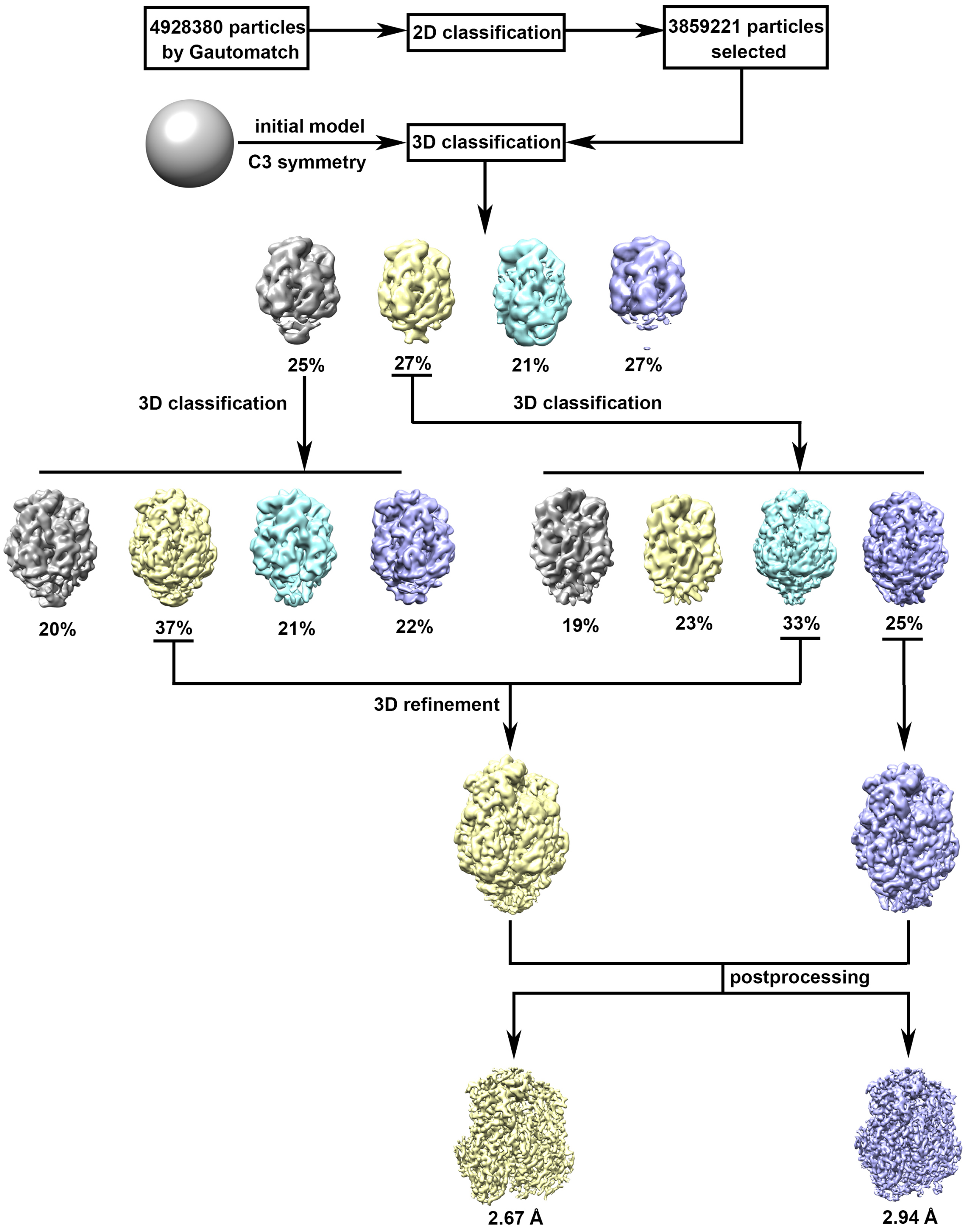
4928380 particles by Gautomatch
2D classification
3859221 particles selected
initial model
C3 symmetry
3D classification
25%
27%
21%
27%
3D classification
3D classification
20%
37%
21%
22%
19%
23%
33%
25%
3D refinement
postprocessing
2.67 Å
2.94 Å

Supplementary information, Figure S2. The flowchart of the p72 data processing procedure. See materials and methods for details.

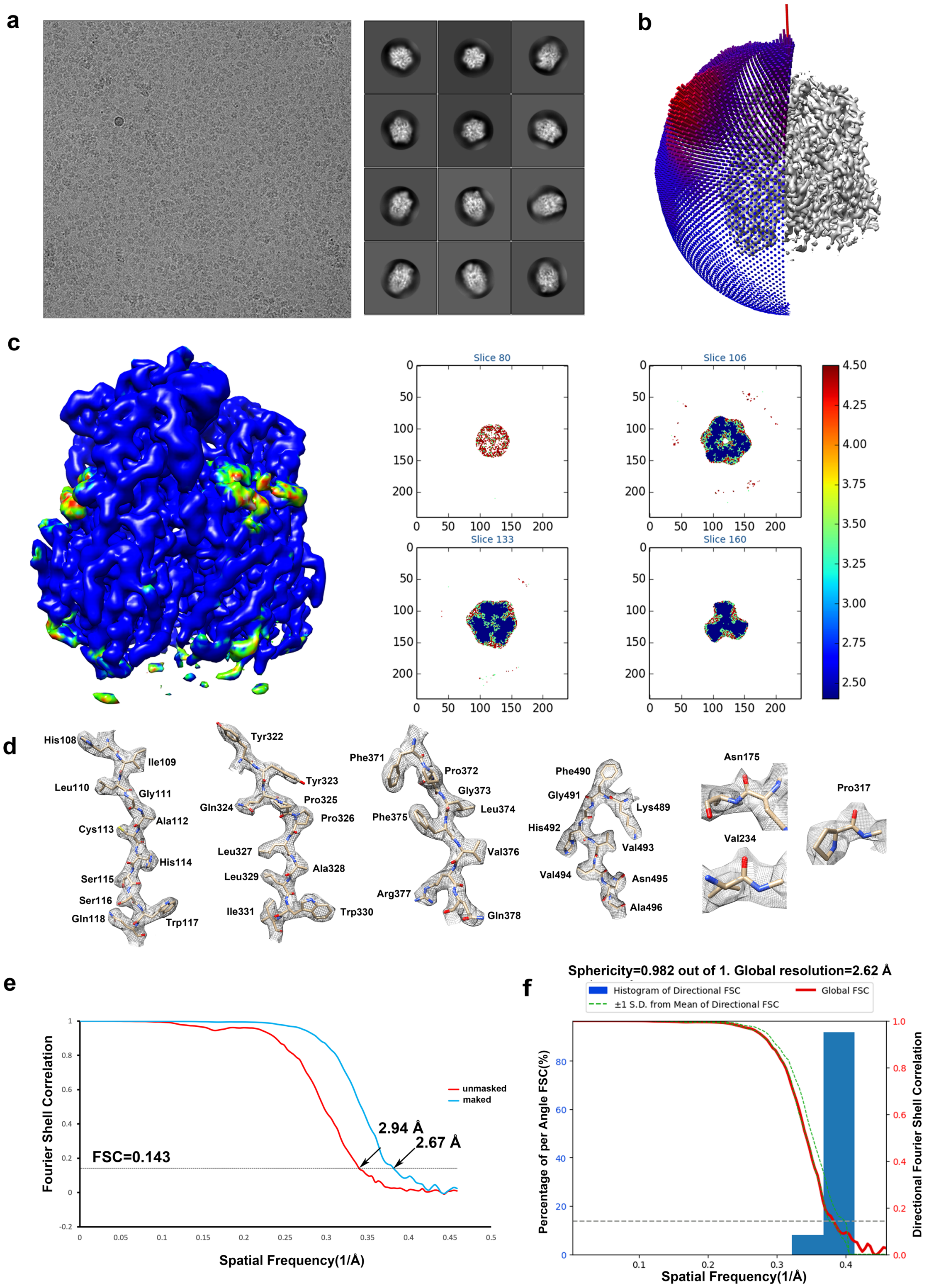
a
b
c
Slice 80
Slice 106
Slice 133
Slice 160
4.50
4.25
4.00
3.75
3.50
3.25
3.00
2.75
2.50
d
His108
Ile109
Leu110
Gly111
Ala112
Cys113
His114
Ser115
Ser116
Gln118
Trp117
Tyr322
Tyr323
Gln324
Pro325
Pro326
Leu327
Ala328
Leu329
Ile331
Trp330
Phe371
Pro372
Gly373
Leu374
Phe375
Val376
Arg377
Gln378
Phe490
Gly491
Lys489
His492
Val493
Val494
Asn495
Ala496
Asn175
Val234
Pro317
e
Fourier Shell Correlation
FSC=0.143
2.94 Å
2.67 Å
unmasked
maked
Spatial Frequency(1/Å)
f
Sphericity=0.982 out of 1. Global resolution=2.62 Å
Histogram of Directional FSC
Global FSC
±1 S.D. from Mean of Directional FSC
Percentage of per Angle FSC(%)
Directional Fourier Shell Correlation
Spatial Frequency(1/Å)

Supplementary information, Figure S3. CryoEM data and reconstruction results of p72. (a) A representative cryoEM micrograph and 2D class averages of p72. (b) Orientation distribution of the particles used for the final refinements of the 2.67 Å map. (c) Local resolution map calculated by using ResMap with the density map that has not been postprocessed. (d) Density maps around the representative regions of the structure. The maps are contoured at 1.3 σ. All the density maps are shown as grep mesh. The models are shown in balls and sticks and the atoms and bonds are colored according to atom type (N: blue, C: tan, S: yellow, O: red). (e) The global Fourier shell correlation curves of the reconstruction. (f) The directional FSC curves of the reconstruction calculated by using the Remote 3DFSC Processing Server[12]. FSC curves for 100 different angular directions were calculated. The global mean FSC is shown in solid red line. The blue bar histogram shows the distribution of the 100 FSC=0.143 values. The FSC curves with FSC=0.143 +1 and -1 standard deviation (S.D.) from the mean are shown in green dash lines.

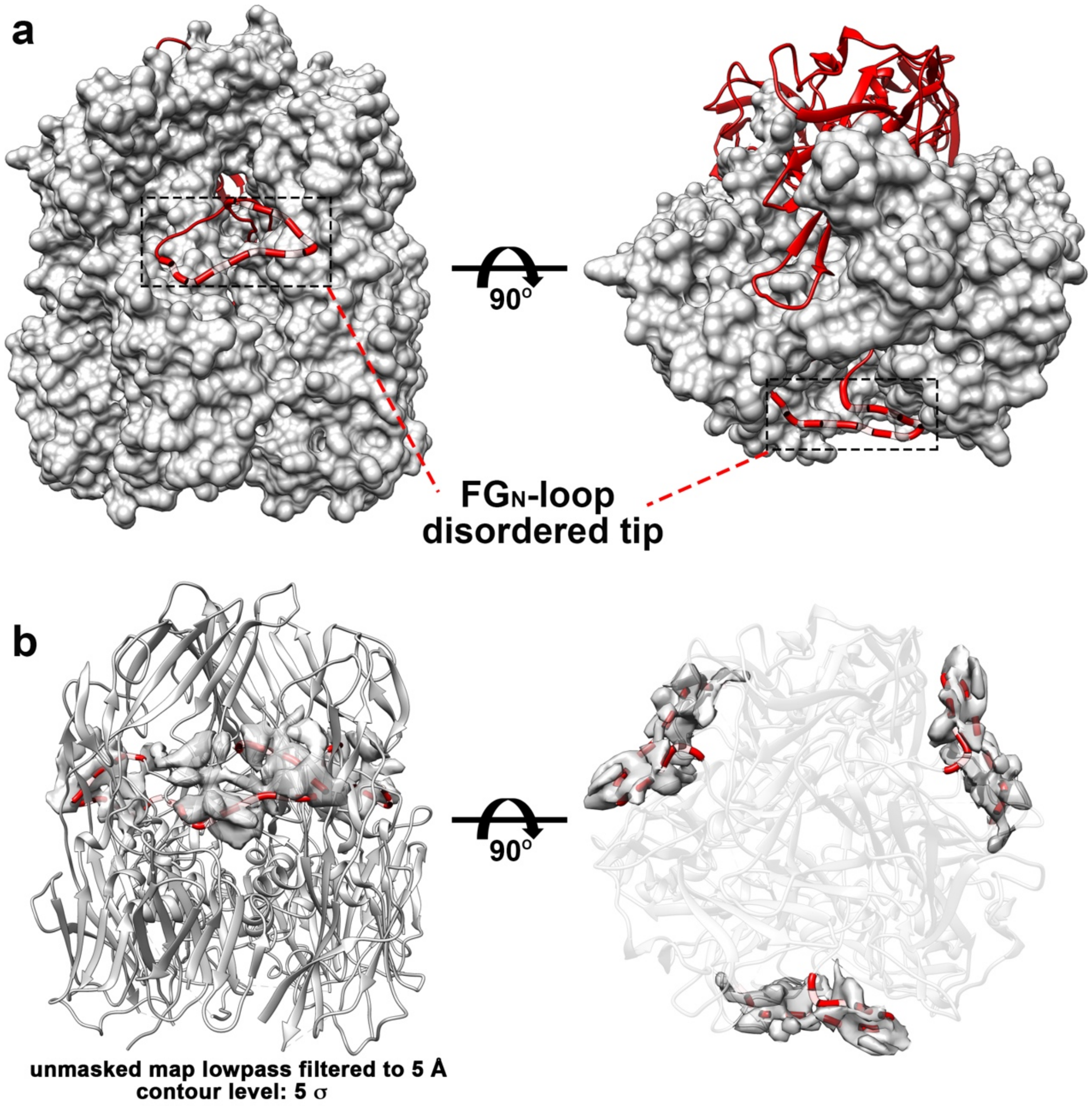
a
90°
FGN-loop
disordered tip
b
90°
unmasked map lowpass filtered to 5 Å
contour level: 5 σ

Supplementary information, Figure S4. The disordered tip of the FG$_N$-loop. (a) Ribbon and surface rendered diagrams showing disordered tip of the FG$_N$-loop located in between the β sheet blades of two neighboring molecules. (b) Densities for the disordered tip are clearly visible when the map is lowpass filtered to 5 Å and contoured at 5 σ.

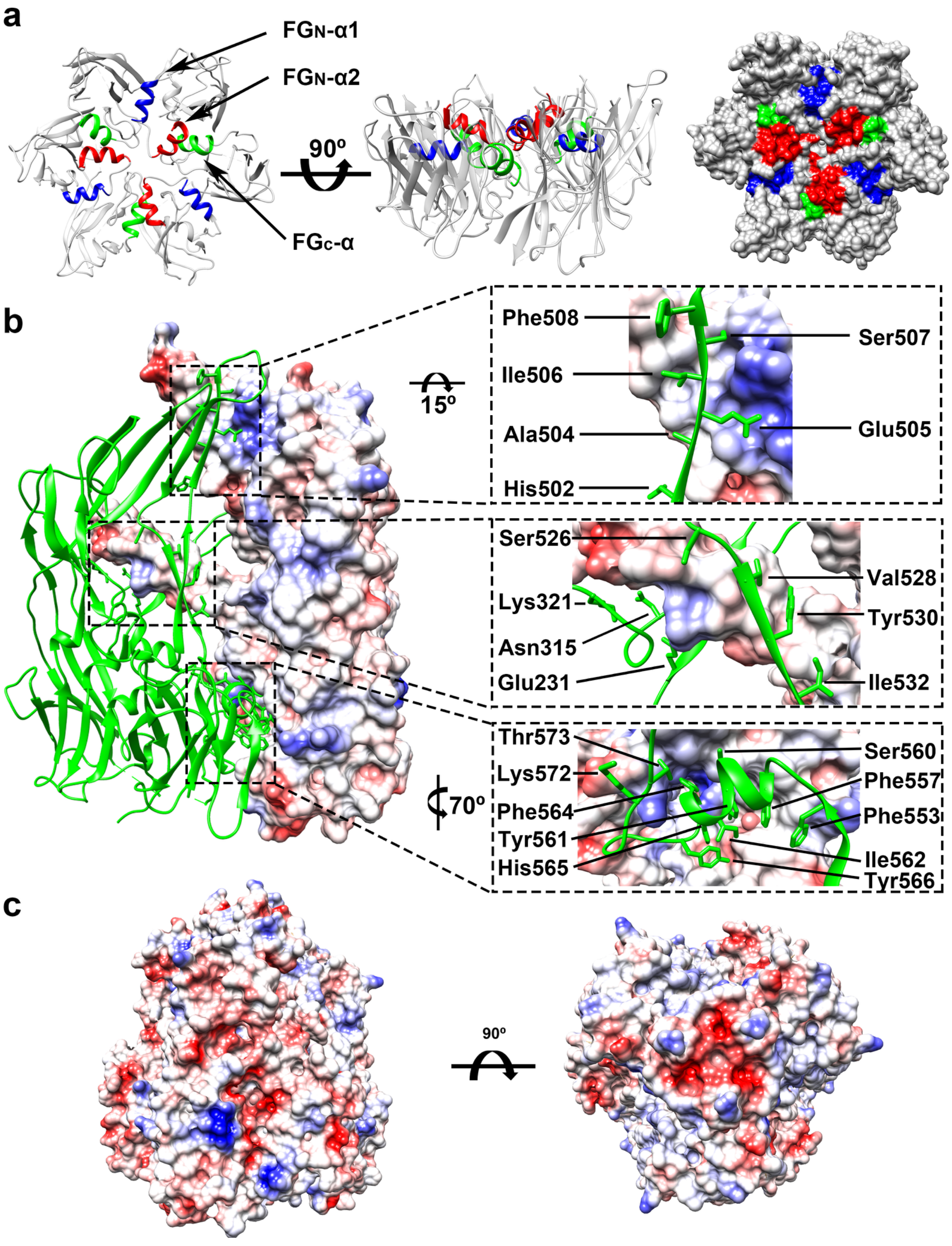
a
FGN-α1
FGN-α2
FGC-α
90º
b
Phe508
Ser507
Ile506
15º
Ala504
Glu505
His502
Ser526
Val528
Lys321
Tyr530
Asn315
Glu231
Ile532
Thr573
Ser560
Lys572
Phe557
70º
Phe564
Phe553
Tyr561
Ile562
His565
Tyr566
c
90º

Supplementary information, Figure S5. The interactions between the monomers of the p72 trimer. (a) Ribbon (top) and surface rendered (bottom) diagrams showing the helix mediated interactions between the jelly-roll barrels. The FG_N-α1 (205-215), FG_N-α2 (221-228) and FG_C-α (557-566) located in the center of the pseudo hexagonal base are colored blue, red and green, respectively. (b) The interaction interface between two protomers. One protomer is shown in surface while the other protomer is shown in ribbon and colored green. The surface is colored according to the surface electrostatic potential with red representing negative electrostatic potential and blue representing positive electrostatic potential. Key residues of one protomer at the interface are shown in balls and sticks. (c) Surface rendered diagrams showing the surface electrostatic potential of the trimer spike. The surface is colored the same as in "b".

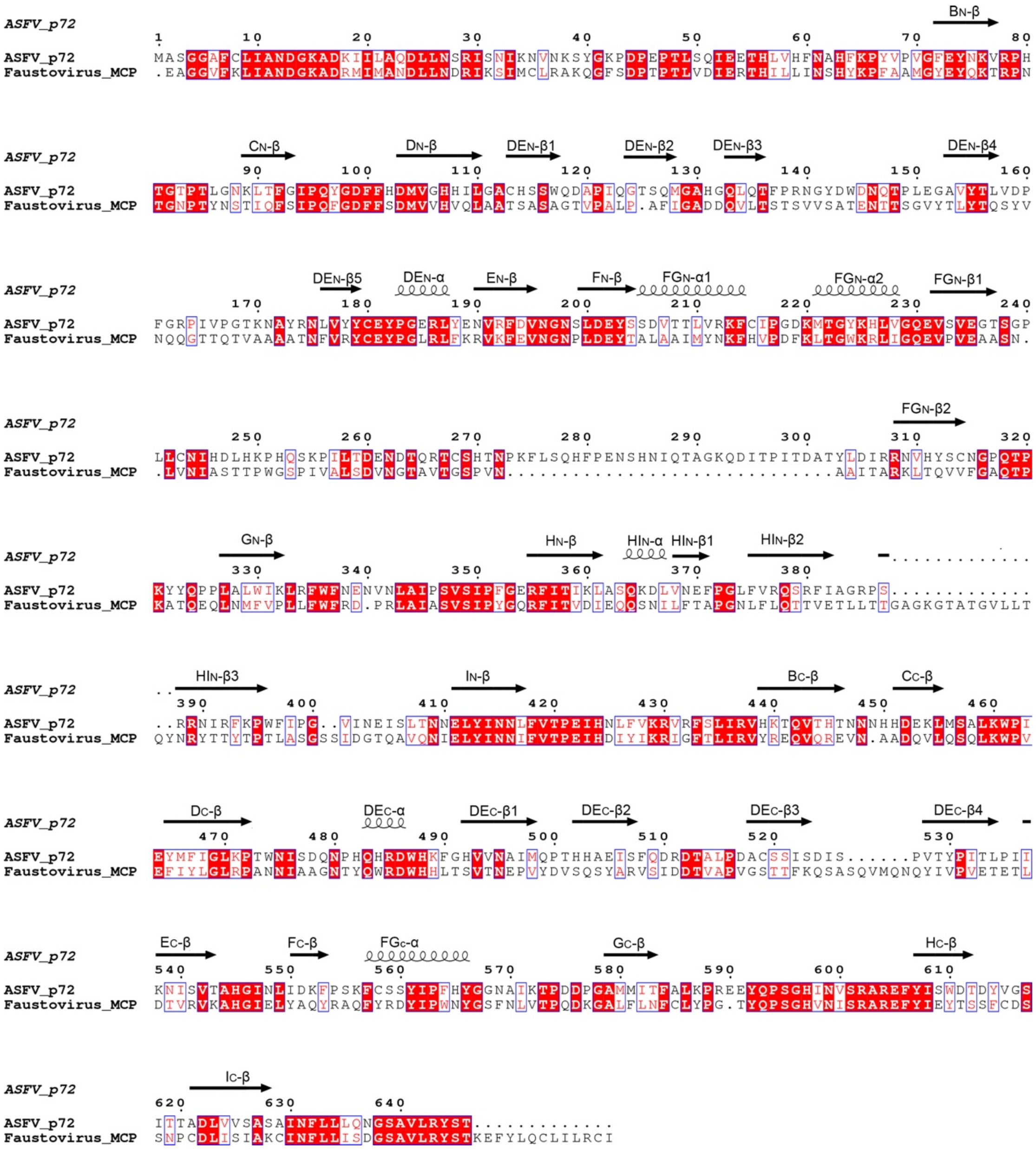
ASFV_p72
Faustovirus_MCP
BN-β
CN-β
DN-β
DEN-β1
DEN-β2
DEN-β3
DEN-β4
DEN-β5
DEN-α
EN-β
FN-β
FGN-α1
FGN-α2
FGN-β1
FGN-β2
GN-β
HN-β
HIN-α
HIN-β1
HIN-β2
HIN-β3
IN-β
BC-β
CC-β
DC-β
DEC-α
DEC-β1
DEC-β2
DEC-β3
DEC-β4
EC-β
FC-β
FGC-α
GC-β
HC-β
IC-β

Supplementary information, Figure S6. Sequence alignments of the ASFV p72 and the faustovirus MCP. Completely conserved residues are boxed and shown in white on a red background. Conserved residues are boxed and shown in red. The secondary structures of p72 are shown on the top of the alignments.

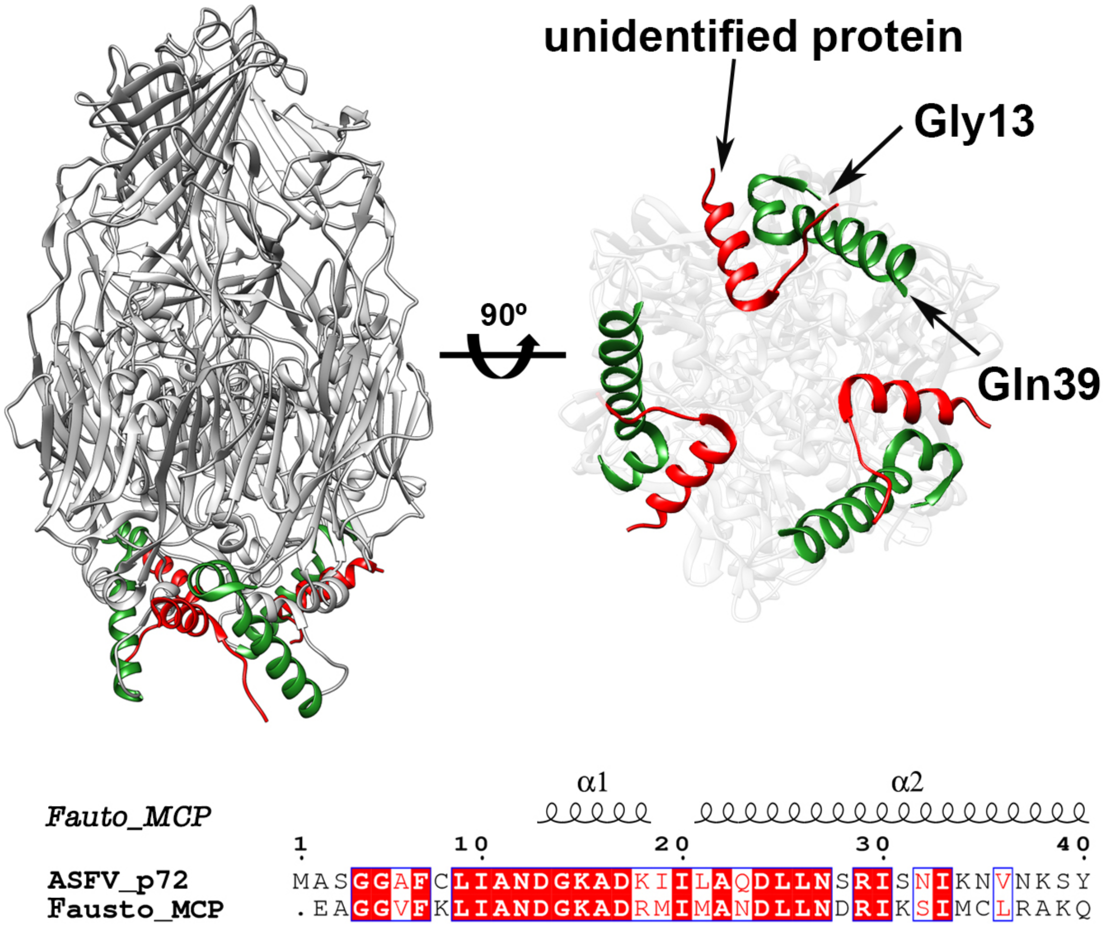
unidentified protein
Gly13
90º
Gln39
α1
α2
Fauto_MCP
1
10
20
30
40
ASFV_p72 MASGGAFCLIANDGKADKIILAQDLLNSRISNIKNVNKSY
Fausto_MCP .EAGGVFKLIANDGKADRMIMANDLLNDRIKSIMCLRAKQ

Supplementary information, Figure S7. Ribbon diagrams showing the helix organization at the bottom of the faustovirus MCP trimer spike. The N-terminal helices of the faustovirus MCP are colored green, which is absent in p72 structure. The helices from the unidentified protein are colored red. Sequence alignments (bottom) showing that 21 residues among 39 residues at the N-terminal regions of the two virus major capsid proteins are identical. The N terminal helix of p72 may become ordered by interaction with a yet unidentified protein during capsid assembling.

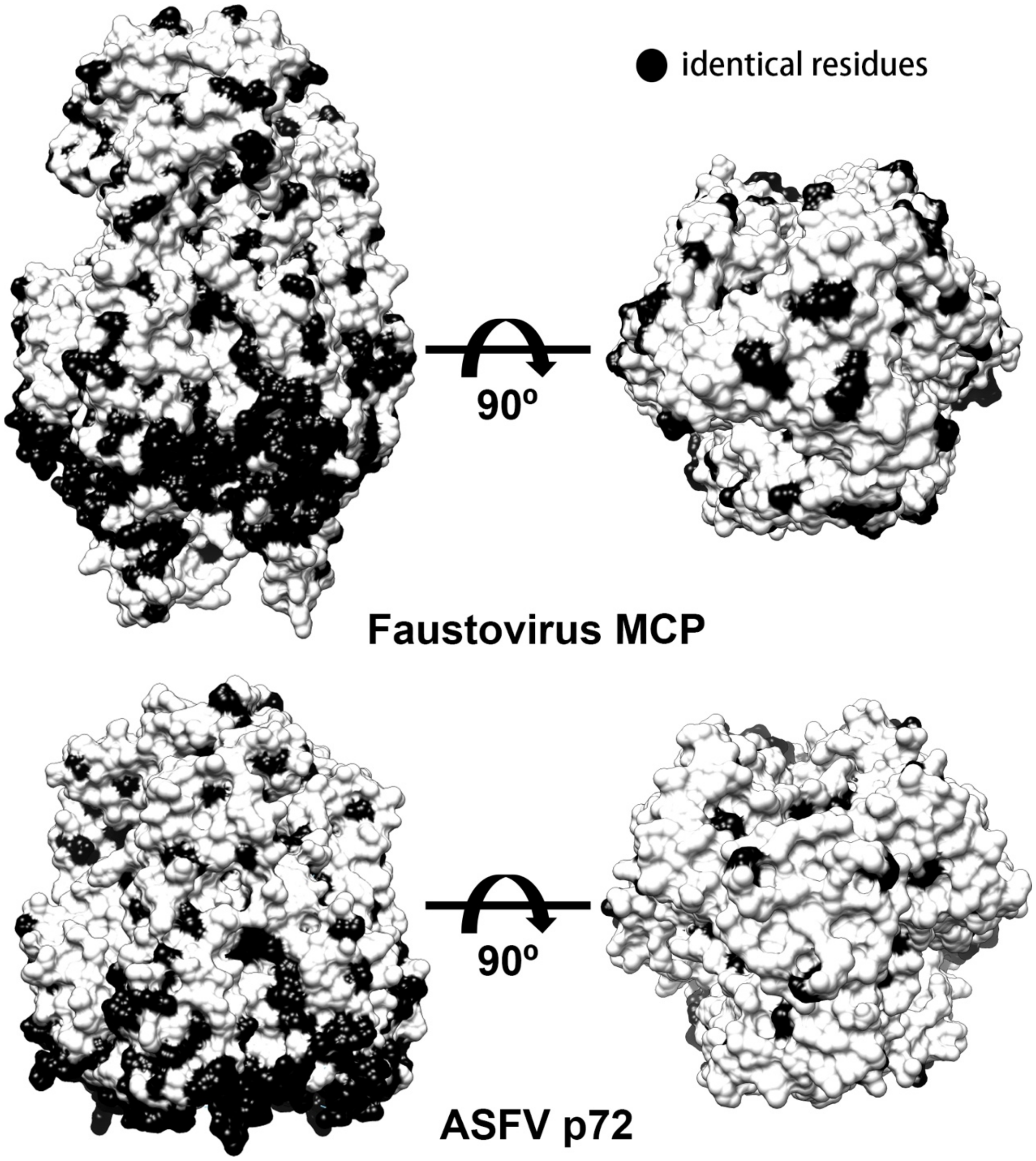
identical residues
90º
Faustovirus MCP
90º
ASFV p72

Supplementary information, Figure S8. Distribution of the conserved residues on the surface of the ASFV p72 and the faustovirus MCP. Surface rendered diagrams showing the distribution of the completely conserved residues on the surface of the ASFV p72 (bottom) and the faustovirus MCP (top). The completely conserved residues between the two virus major capsid proteins are colored black and are mostly located in the pseudo hexagonal base of the spike.

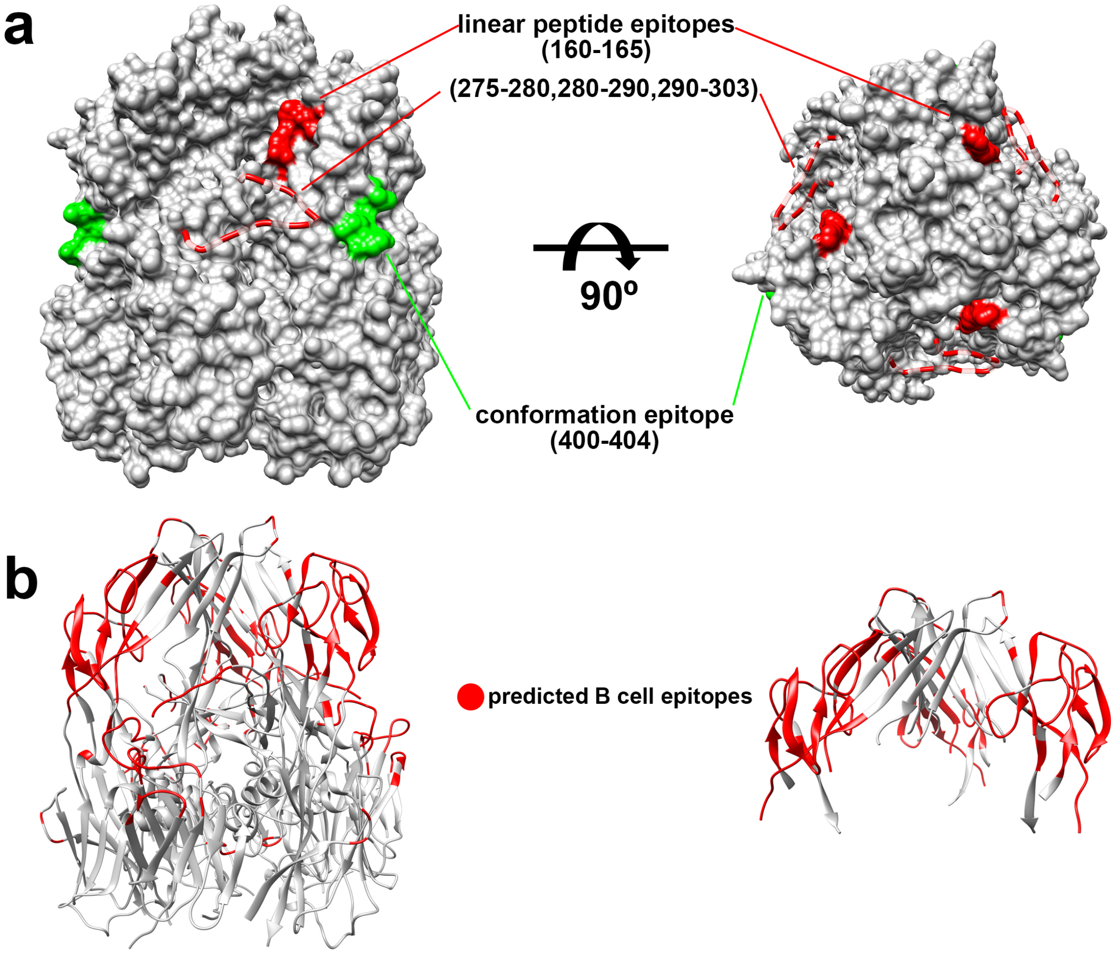

a
linear peptide epitopes
(160-165)
(275-280,280-290,290-303)
90º
conformation epitope
(400-404)
b
predicted B cell epitopes

Supplementary information, Figure S9. Location of identified epitopes on the p72 trimer spike. (a) The identified linear (red) and conformational (green) epitopes on the surface of p72. The disordered 249-303 loop at the tip of the insertion FG_N is shown in red dash line. (b) The predicted B cell epitopes (colored red) of p72. The prediction was performed by using DiscoTope 2.0 (http://www.cbs.dtu.dk/services/DiscoTope/)[16] with a threshold set to -3.7, a default value suitable for most of the predictions. The prediction is based on the statistics, spatial information, and surface accessibility of the amino acids.

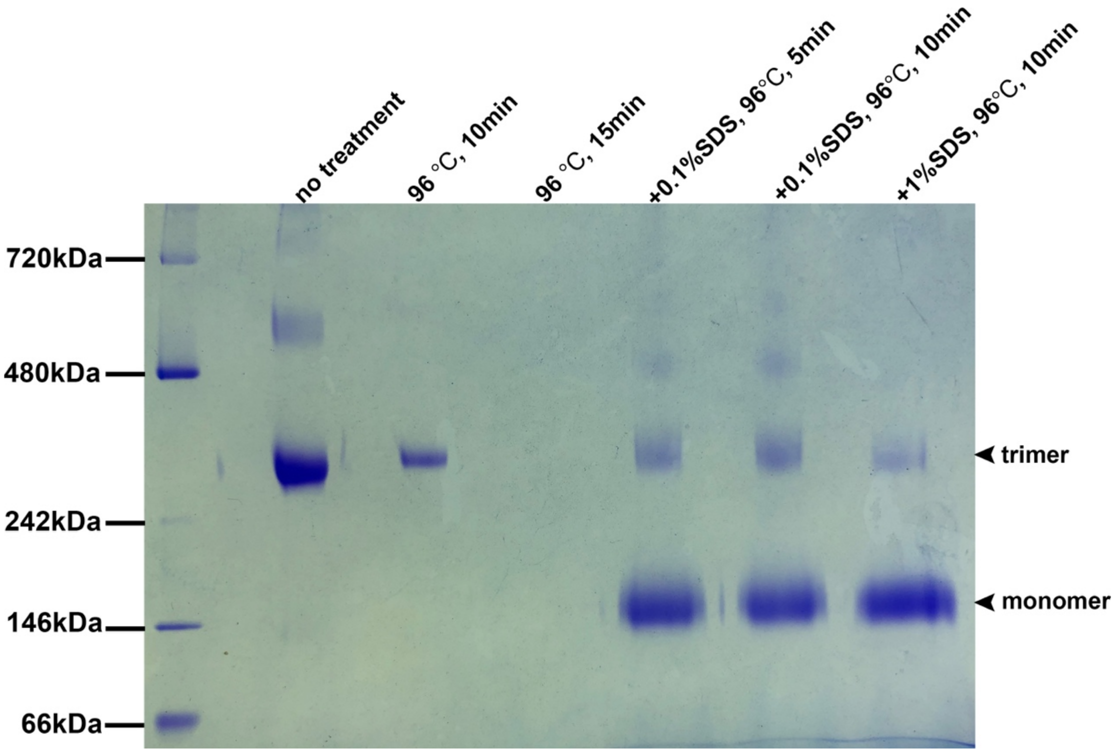
no treatment
96 °C, 10min
96 °C, 15min
+0.1%SDS, 96°C, 5min
+0.1%SDS, 96°C, 10min
+1%SDS, 96°C, 10min
720kDa
480kDa
242kDa
146kDa
66kDa
trimer
monomer

Supplementary information, Figure S10. Native page gel analysis of the heat treated p72. The p72 protein was treated at 96 °C with or without SDS. The positions of the p72 monomer and trimer are indicated.

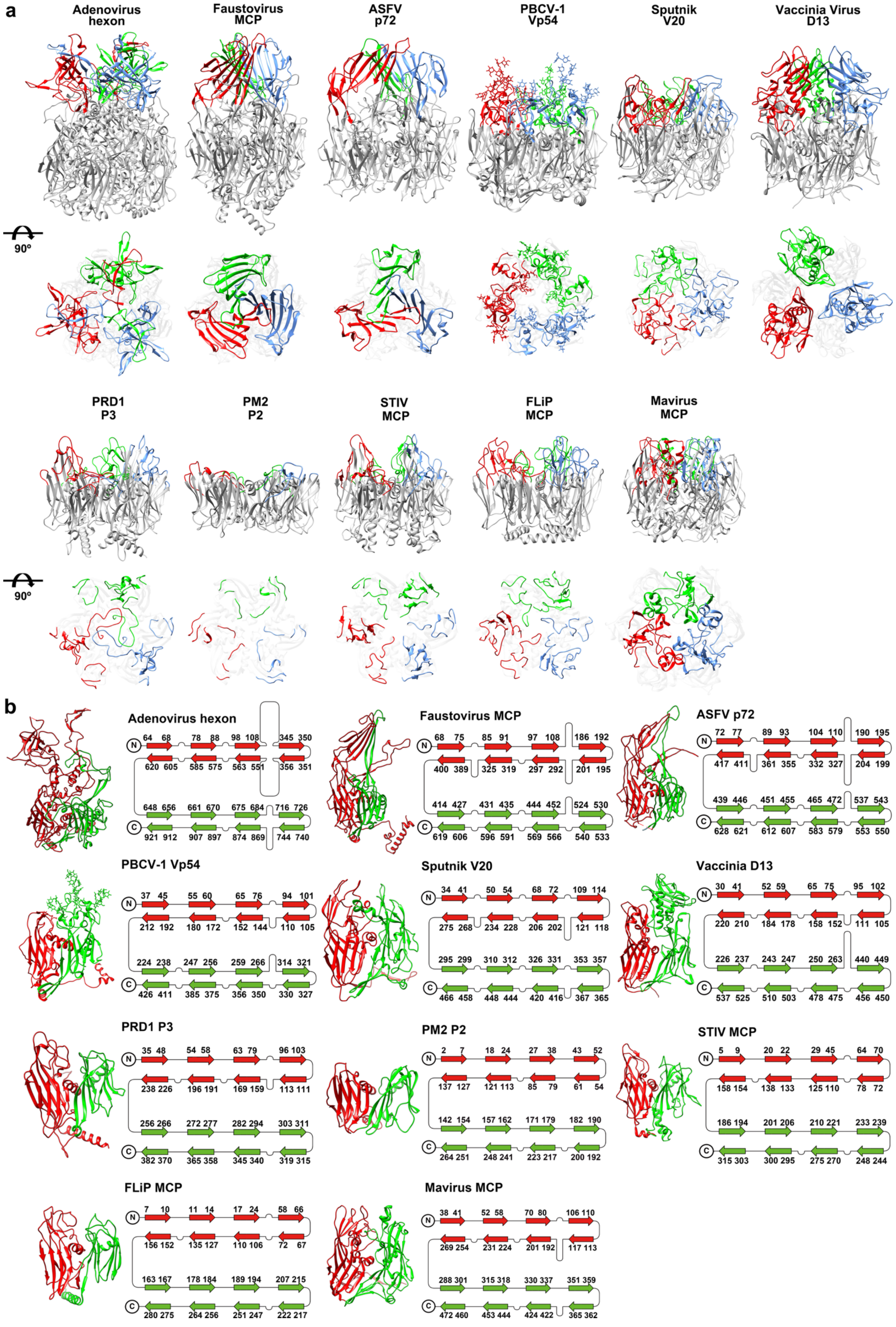

a
Adenovirus hexon
Faustovirus MCP
ASFV p72
PBCV-1 Vp54
Sputnik V20
Vaccinia Virus D13
90º
PRD1 P3
PM2 P2
STIV MCP
FLiP MCP
Mavirus MCP
90º
b
Adenovirus hexon
N 64 68 78 88 98 108 345 350
620 605 585 575 563 551 356 351
648 656 661 670 675 684 716 726
C 921 912 907 897 874 869 744 740
Faustovirus MCP
N 68 75 85 91 97 108 186 192
400 389 325 319 297 292 201 195
414 427 431 435 444 452 524 530
C 619 606 596 591 569 566 540 533
ASFV p72
N 72 77 89 93 104 110 190 195
417 411 361 355 332 327 204 199
439 446 451 455 465 472 537 543
C 628 621 612 607 583 579 553 550
PBCV-1 Vp54
N 37 45 55 60 65 76 94 101
212 192 180 172 152 144 110 105
224 238 247 256 259 266 314 321
C 426 411 385 375 356 350 330 327
Sputnik V20
N 34 41 50 54 68 72 109 114
275 268 234 228 206 202 121 118
295 299 310 312 326 331 353 357
C 466 458 448 444 420 416 367 365
Vaccinia D13
N 30 41 52 59 65 75 95 102
220 210 184 178 158 152 111 105
226 237 243 247 250 263 440 449
C 537 525 510 503 478 475 456 450
PRD1 P3
N 35 48 54 58 63 79 96 103
238 226 196 191 169 159 113 111
256 266 272 277 282 294 303 311
C 382 370 365 358 345 340 319 315
PM2 P2
N 2 7 18 24 27 38 43 52
137 127 121 113 85 79 61 54
142 154 157 162 171 179 182 190
C 264 251 248 241 223 217 200 192
STIV MCP
N 5 9 20 22 29 45 64 70
158 154 138 133 125 110 78 72
186 194 201 206 210 221 233 239
C 315 303 300 295 275 270 248 244
FLiP MCP
N 7 10 11 14 17 24 58 66
156 152 135 127 110 106 72 67
163 167 178 184 189 194 207 215
C 280 275 264 256 251 247 222 217
Mavirus MCP
N 38 41 52 58 70 80 106 110
269 254 231 224 201 192 117 113
288 301 315 318 330 337 351 359
C 472 460 453 444 424 422 365 362

Supplementary information, Figure S11. Structure comparisons of the jelly-roll viral capsid or scaffold proteins. (a) Ribbon diagrams showing the architectures of the double jelly-roll viral capsid proteins and scaffold protein, including the adenovirus hexon (PDB ID: 6B1T), faustovirus MCP (PDB ID: 5J7O), PCVB-1 Vp54 (PDB ID: 5TIP), Sputnik V20 (PDB ID: 3J26), Vaccinia virus scaffold protein D13 (PDB ID: 2YGB), PRD1 P3 (PDB ID: 1CJD), PM2 P2 (PDB ID: 2VVF), STIV MCP (PDB ID: 2BBD), FLiP MCP (PDB ID: 5OAC), Mavirus MCP (PDB ID: 6G45). The pseudo hexagonal bases are colored gray. The three insertion crown domains are colored red, green, and cornflower blue, respectively. (b) Structure comparisons of the double jelly-roll viral capsid or scaffold proteins. The structure and topology of a monomer are shown. The N- and C-terminal jelly-roll domains are colored red and green, respectively

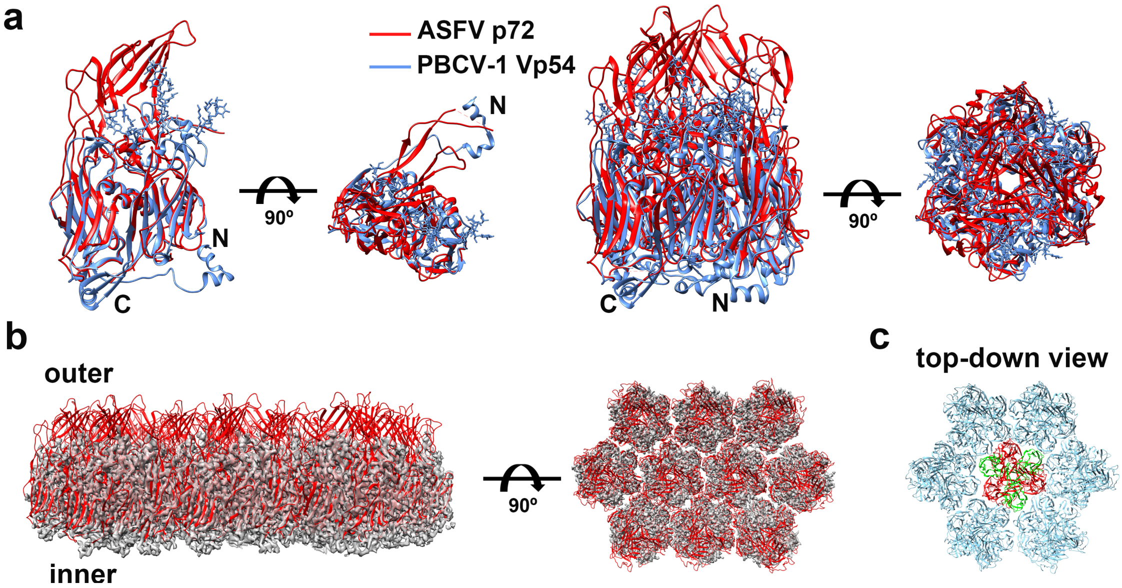
a
ASFV p72
PBCV-1 Vp54
N
C
90º
N
C
N
90º
b
outer
inner
90º
c
top-down view

Supplementary information, Figure S12. Structure of the pseudo p72 capsid assembly generated based on the PBCV-1 structure. (a) Structure superposition of the ASFV p72 (red) and the PBCV-1 Vp54 (cornflower blue). (b) The fitted p72 trimers (red ribbons) in the cryoEM map of the PBCV-1 capsid (EMD-7626). The map is contoured at 2 σ. The PBCV-1 capsid does not have the top protruding insertion domains as has the ASFV p72. (c) The fitted p72 trimer spikes show a zigzag arrangement of their pseudo hexagonal bases.

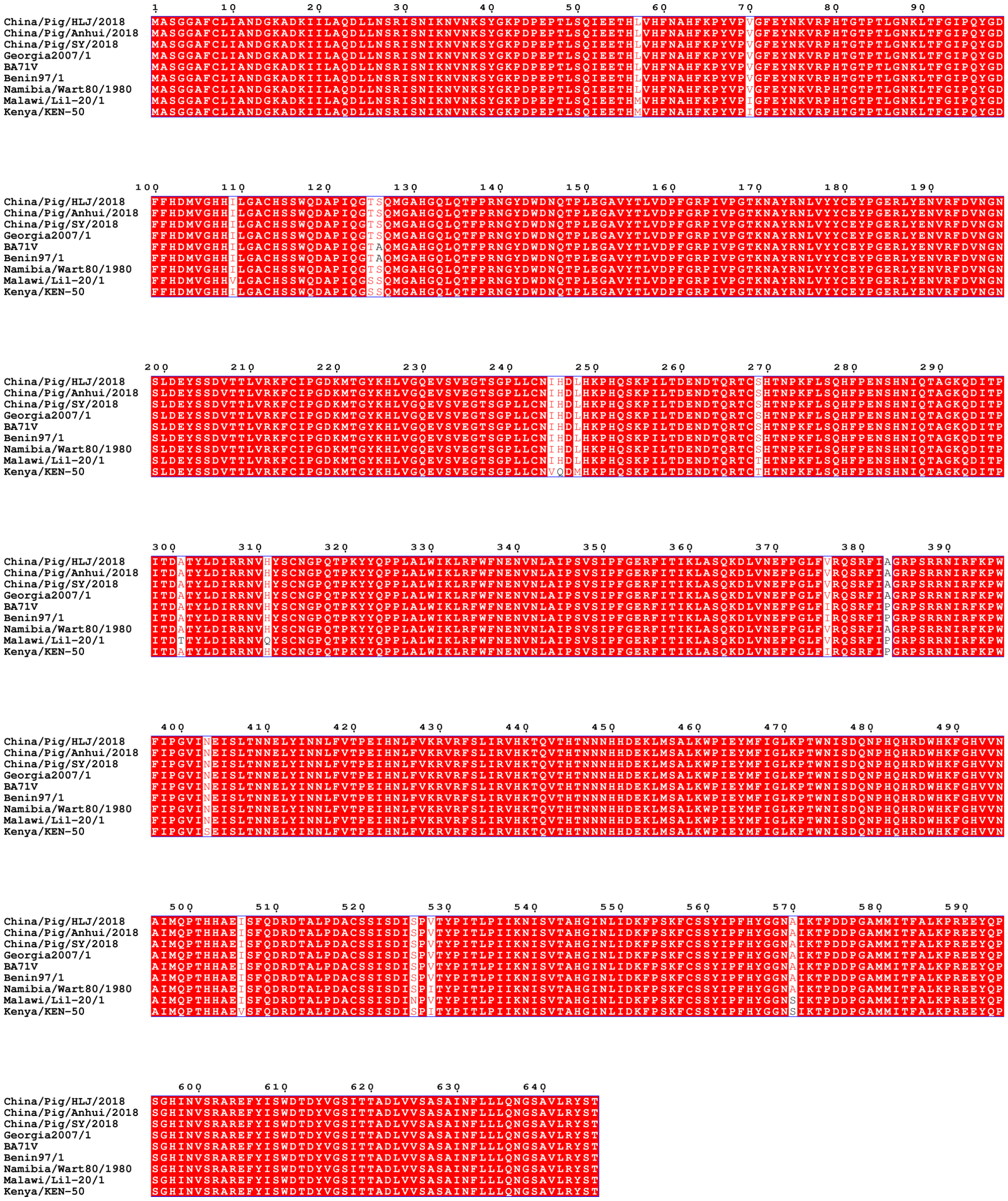
China/Pig/HLJ/2018
China/Pig/Anhui/2018
China/Pig/SY/2018
Georgia2007/1
BA71V
Benin97/1
Namibia/Wart80/1980
Malawi/Lil-20/1
Kenya/KEN-50

Supplementary information, Figure S13. Sequence alignments of the p72s from different ASFV isolates. China/Pig/HLJ/2018 (GenBank: QBH90570.1), China/Pig/Anhui/2018 (GenBank: AYW34053.1), China/Pig/SY/2018 (GenBank: AXP99042.1), Georgia/2007/1 (GenBank: CBW46748.1), BA71V (GenBankL: P22776.2), Benin97/1 (GenBank: CAN10181.1), Namibia/Wart80/1980 (GenBank: Q5IZI7.1), Malavi/Lil-20/1 (GenBank: Q8V9S6.1), Kenya/Ken-50 (GenBank: Q5IZJ5.1), Completely conserved residues are boxed and shown in white on a red background.

Supplementary information, Table S1. Cryo-EM data collection and image processing statistics

Sample	p72 trimer spike
Acceleration voltage (keV)	300
Detector	K2 Summit
Super-resolution pixel size (Å)	0.5455
Particles of final refinement	693388
Resolution (Å)	2.67
B-factor ($Å^2$)	-104
R.m.s. deviations (bond lengths) (Å)	0.005
R.m.s. deviations (bond angles) (°)	0.931
Correlation coefficient (CCmask)	0.86
Rotamer outliers (%)	4.86
Clashscore	2.64
Ramachandran favored (%)	92.9
Ramachandran allowed(%)	7.1
Ramachandran outliers (%)	0.0
EMRinger Score	3.74
PDB ID	6KU9
EMDB ID	EMD-0776

Supplementary information, Table S2. Structure comparisons of the double jelly-roll viral proteins

Structure	PDB ID	R.M.S.D.*
Adenovirus Hexon	6B1T	3.29 Å
faustovirus MCP	5J7O	1.25 Å
PBCV-1 Vp54	5TIP	1.53 Å
Sputnik V20	3J26	2.30 Å
Vaccinia virus D13	2YGB	2.04 Å
PRD1 P3	1CJD	2.70 Å
PM2 P2	2VVF	2.64 Å
STIV MCP	2BBD	2.67 Å
FLiP MCP	5OAC	2.79 Å
Mavirus MCP	6G45	2.38 Å

* only the C_α atoms of the jelly-roll domains were used for the R.M.S.D. calculation.

References

1 Bastos AD, Penrith ML, Cruciere C *et al.* Genotyping field strains of African swine fever virus by partial p72 gene characterisation. *Arch Virol* 2003; **148**:693-706.

2 Zhao D, Liu R, Zhang X *et al.* Replication and virulence in pigs of the first African swine fever virus isolated in China. *Emerg Microbes Infect* 2019; **8**:438-447.

3 Klose T, Reteno DG, Benamar S *et al.* Structure of faustovirus, a large dsDNA virus. *Proceedings of the National Academy of Sciences* 2016; **113**:6206-6211.

4 Constance L. Cepko PAS. assembly of adenovirus major capsid protein is mediated by a nonvirion protein. *Cell* 1982; **31**:407-415.

5 Lei JL, Frank J. Automated acquisition of cryo-electron micrographs for single particle reconstruction on an FEI Tecnai electron microscope. *J Struct Biol* 2005; **150**:69-80.

6 Zheng SQ, Palovcak E, Armache J-P, Verba KA, Cheng Y, Agard DA. MotionCor2: anisotropic correction of beam-induced motion for improved cryo-electron microscopy. *Nature Methods* 2017; **14**:331-332.

7 Zhang K. Gctf: Real-time CTF determination and correction. *J Struct Biol* 2016; **193**:1-12.

8 Zivanov J, Nakane T, Forsberg BO *et al.* New tools for automated high-resolution cryo-EM structure determination in RELION-3. *eLife* 2018; **7**.

9 van Heel M, Schatz M. Fourier shell correlation threshold criteria. *Journal of Structural Biology* 2005; **151**:250-262.

10 Scheres SH, Chen S. Prevention of overfitting in cryo-EM structure determination. *Nat Methods* 2012; **9**:853-854.

11 Kucukelbir A, Sigworth FJ, Tagare HD. Quantifying the local resolution of cryo-EM density maps. *Nat Methods* 2014; **11**:63-65.

12 Tan YZ, Baldwin PR, Davis JH *et al.* Addressing preferred specimen orientation in single-particle cryo-EM through tilting. *Nat Methods* 2017; **14**:793-796.

13 Emsley P, Lohkamp B, Scott WG, Cowtan K. Features and development of Coot. *Acta Crystallogr D Biol Crystallogr* 2010; **66**:486-501.

14 Adams PD, Afonine PV, Bunkoczi G *et al.* PHENIX: a comprehensive Python-based system for macromolecular structure solution. *Acta Crystallogr D Biol Crystallogr* 2010; **66**:213-221.

15 Holm L. Benchmarking Fold Detection by DaliLite v.5. *Bioinformatics* 2019.

16 Kringelum JV, Lundegaard C, Lund O, Nielsen M. Reliable B cell epitope predictions: impacts of method development and improved benchmarking. *PLoS Comput Biol* 2012; **8**:e1002829.
